# Barriers to Pediatric Rheumatology Care: A Survey of Pediatric Clinical Practices at Sheikh Khalifa Medical City in Abu Dhabi, United Arab Emirates

**DOI:** 10.7759/cureus.70531

**Published:** 2024-09-30

**Authors:** Ayaan Khan, Kamran Mahmood

**Affiliations:** 1 Pediatric Rheumatology, Sheikh Khalifa Medical City Abu Dhabi, Abu Dhabi, ARE

**Keywords:** access to care, clinical care, examination, pediatric rheumatology, training

## Abstract

Background

Access to pediatric rheumatology (PR) is limited in Abu Dhabi, United Arab Emirates, with only three trained pediatric rheumatologists in Abu Dhabi. However, the limited access and current levels of PR care in the Abu Dhabi workforce have not yet been formally assessed. The objective of this study was to evaluate the confidence in the delivery of PR patient care and the need for a structured PR training program among pediatricians.

Methods

The anonymous web-based Google Forms survey (Google LLC, Mountain View, CA, USA) included 16 items on PR training programs and patient care. The survey was sent to physicians in the pediatric department in Sheikh Khalifa Medical City, Abu Dhabi, through June 2024.

Results

Among the 54 participants, the majority were general pediatricians (70.4%). Most participants encountered PR patients monthly (51.9%) or weekly (29.6%), yet 87% had less than one year of experience in PR. Confidence levels in examining, investigating, and managing PR patients were generally low to moderate, while physicians primarily reported high confidence in referring patients. A significant need for a structured PR training program at SKMC was identified, with 92.6% of participants advocating for its implementation.

Conclusions

The majority of pediatricians do not feel confident in examining, investigating, and managing pediatric patients with rheumatic diseases. There is a need for a structured PR program at Sheikh Khalifa Medical City, Abu Dhabi, due to a significant shortage in PR physicians.

## Introduction

Pediatric rheumatology (PR) is a subspecialization of pediatrics, and it pertains to illnesses related to a wide range of autoinflammatory and autoimmune problems. The most notable pediatric rheumatic diseases include juvenile idiopathic arthritis, followed by juvenile-onset systemic lupus erythematosus [1]. They have a substantial impact on children's health and well-being. Approximately 6-7 million children worldwide suffer from PR diseases yearly [2]. The PR workforce aims to provide children with access to superior clinical care; however, this is lacking due to a shortage of the PR workforce itself. The limited access to PR care worldwide is especially true in the Middle East: the proportion of pediatric rheumatologists is 6.7 per million of the population. Moreover, there are only three trained pediatric rheumatologists in Abu Dhabi, UAE, and only two tertiary PR services are available, making access to specialized PR care exceedingly limited [3]. This lack of expert care can cause two main challenges, resulting in lower outcomes for children with rheumatic disorders. Firstly, PR patients are, the majority of the time, treated by primary care providers, at least initially, who have an inadequate awareness of PR diseases and conditions. Secondly, if children are referred to the few PR physicians available, there is a severe delay in diagnosis and treatment. As these diseases are progressive in nature, an obstruction to the initiation of treatment causes drastic impacts on the prognosis of patients [4]. Despite this deficit, the degree and impact of limited access to PR treatment have not been fully assessed. The goal of this study was to examine pediatricians' confidence in providing PR patient care and determine the necessity for a structured PR training program at Sheikh Khalifa Medical City (SKMC), Abu Dhabi.

## Materials and methods

Survey development and distribution

This cross-sectional study was conducted through an anonymous web-based self-administered poll created utilizing Google Forms (Google LLC, Mountain View, CA, USA) to assess PR training programs and patient care. Face validity was determined by a panel of five pediatricians who examined the questionnaire for its clarity, format, ease of comprehension, and overall presentation. To ensure content validity, a PR consultant reviewed the survey, assessing its thoroughness and relevance to PR knowledge and training and agreeing on the key questions to be included in the final questionnaire. A pilot test was conducted with a small sample size from the pediatric department prior to the full distribution of the survey. The sampling technique used for this study was quota sampling, as the survey was sent to all physicians solely in SKMC's pediatric department in June 2024, including consultants, specialists, and residents. Aside from those who did not work within the pediatric department or who were on leave during the survey distribution period, the study had no exclusion criteria to ensure a comprehensive representation of the department's physicians. The study complied with ethical considerations, and thus the participants who were invited by email were guaranteed confidentiality and anonymity. The Institutional Review Board of Sheikh Khalifa Medical City approved the study (approval number: REC-30.04.2024 (RS-859)).

Survey content

The poll consisted of 16 questions addressing personal information, professional history, confidence levels in various elements of PR treatment, and thoughts on the necessity for a PR training program at SKMC. Informed consent was required as a prerequisite for participation, and no identification data such as subject names were collected. The survey asked questions about gender, age, country of training, position, speciality, years of experience in pediatrics and PR, frequency of encountering PR patients, confidence in examining, investigating, managing, and referring patients, and whether past workplaces offered PR training programs. The questions were a mix of closed-ended questions and Likert scale items. The survey took an average of five minutes to complete.

Data analysis

The collected descriptive data was managed and analyzed using Microsoft Excel (Microsoft Corporation, Redmond, WA, USA) and Google Spreadsheets (Google LLC, Mountain View, CA, USA). The categorical data, such as gender, training location, professional role, and speciality, were calculated and represented through frequencies and percentages. Descriptive statistics such as means and standard deviations were used to analyze and present Likert scale responses, reflecting the confidence levels in examining, managing, and referring to PR patients. Statistical significance was set at p<0.05, and data was collected by only the research team members. Access to the research data was confined solely to the study team members.

## Results

Participant demographics and professional characteristics

A total of 54 participants completed the survey. The demographic breakdown was as follows: 68.5% (37/54) were female and 31.5% (17/54) were male (Table 1). Regarding age, 16.7% were 50 years old or older, 37% were 40-49 years old, 13.1% were 30-39 years old, and 22.2% were under 30 years old (Table 1). In terms of residency training, 44.4% were trained in the UAE, with the remainder trained in various countries including the UK, USA, Pakistan, India, Egypt, Canada, Europe, Yemen, and Jordan (Table 1). Participants held positions as consultants (38.9%), specialists (37%), and residents (24.1%) (Table 2). The majority (70.4%) were general pediatricians, with smaller proportions representing various pediatric subspecialties (Table 2). The distribution of years of experience in pediatrics varied, with 24% having one to five years, 16.7% having six to 10 years, 16.7% having 11-15 years, 16.7% having 16-20 years, 11.1% having 21-25 years, 7.4% having over 25 years, and 5.6% having less than one year (Table 2). However, the majority (87%) had less than one year of experience in PR (Table 2).

**Table 1:** Demographic characteristics of participants UAE: United Arab Emirates, UK: United Kingdom, US: United States

	Characteristic	Total (N = 54), n (%)
Gender	Male	37 (68.5)
	Female	17 (31.5)
Age	50+ years	9 (16.7)
	40-49 years	20 (37)
	30-39 years	13 (13.1)
	Under 30 years	12 (22.2)
Residency training location	UAE	24 (44.4)
	UK	9 (16.7)
	US	6 (11.1)
	Pakistan	2 (3.7)
	India	4 (7.4)
	Egypt	3 (5.6)
	Canada	2 (3.7)
	Rest of Europe	2 (3.7)
	Yemen	1 (1.9)
	Jordan	1 (1.9)

**Table 2:** Professional characteristics of participants PR: pediatric rheumatology

	Characteristic	Total (N = 54), n (%)
Position	Consultant	21 (38.9)
	Specialist	20 (37)
	Resident	13 (24.1)
Speciality	General pediatrics	38 (70.4)
	PR	2 (3.7)
	Pediatric geneticists	3 (5.6)
	Pediatric pulmonologists	2 (3.7)
	Pediatric hematologists/oncologists	3 (5.6)
	Pediatric gastroenterologists	3 (5.6)
	Pediatric nephrologists	3 (5.6)
Years of experience in pediatrics	25+ years	4 (7.4)
	21-25 years	6 (11.1)
	16-20 years	9 (16.7)
	11-15 years	9 (16.7)
	6-10 years	9 (16.7)
	1-5 years	23 (24)
	Less than 1 year	3 (5.6)
Years of experience in PR	25+ years	0 (0)
	21-25 years	0 (0)
	16-20 years	0 (0)
	11-15 years	1 (1.9)
	6-10 years	1 (1.9)
	1-5 years	5 (9.3)
	Less than 1 year	47 (87)

PR awareness and clinical care delivery

In terms of patient encounters, 1.9% saw PR patients daily, 29.6% weekly, 51.9% monthly, and 16.7% yearly (Table 3). Confidence levels (from 1 to 5, with 1 being the lowest confidence and 5 being the highest confidence) in examining PR patients were low, with 7.4% reporting a confidence level of 1, 1.9% a confidence level of 2, 50% a confidence level of 3, 38.9% a confidence level of 4, and only 1.9% a confidence level of 5 (Table 3). Confidence in investigating and managing PR patients was similarly low, with 5.6% reporting a confidence level of 1, 25.9% a confidence level of 2, 46.3% a confidence level of 3, 20.4% a confidence level of 4, and 1.9% a confidence level of 5 (Table 3). However, confidence in referring PR patients was higher, with no participants reporting a confidence level of 1, 1.9% a confidence level of 2, 11.1% a confidence level of 3, 37% a confidence level of 4, and 50% a confidence level of 5 (Table 3).

**Table 3:** PR patients and confidence levels PR: pediatric rheumatology

	Characteristic	Total (N = 54), n (%)
Frequency of encountering PR patients	Daily	1 (1.9)
	Weekly	16 (29.6)
	Monthly	28 (51.9)
	Yearly	9 (16.7)
Confidence in examining PR patients	Low (1-2)	5 (9.3)
	Moderate (3)	27 (50)
	High (4-5)	22 (40.8)
Confidence in investigating and managing PR patients	Low (1-2)	17 (31.5)
	Moderate (3)	25 (46.3)
	High (4-5)	12 (22.3)
Confidence in referring PR patients	Low (1-2)	1 (1.9)
	Moderate (3)	6 (11.1)
	High (4-5)	47 (87)

PR training

Regarding past workplaces offering PR training programs, 46.3% of participants had access to a PR training program at past workplaces, while 53.7% did not (Table 4). A significant majority (92.6%) expressed the need for a PR training program at SKMC, as 77% of residents who participated in the survey indicated willingness to participate in such a program (Table 4). However, out of the total 13 residents in the study, 23% of resident participants were not willing to participate in a potential PR program (Table 4).

**Table 4:** PR training PR: pediatric rheumatology, SKMC: Sheikh Khalifa Medical City

	Characteristic	Total (N = 54), n (%)
Past workplaces offering PR training	Yes	25 (46.3)
	No	29 (53.7)
Need for PR training program at SKMC	Yes	50 (92.6)
	No	4 (7.4)
Residents willing to participate in PR training (n=13)	Yes	10 (77)
	No	3 (23)

## Discussion

This is the first study in Abu Dhabi assessing the barriers to PR clinic manifestations. The findings of this survey reveal significant disparities in the confidence and preparedness of physicians at SKMC in addressing pediatric rheumatic cases. The majority of respondents had limited expertise with PR, as seen by their low to moderate confidence in examining, investigating, and managing PR patients. High confidence in referring patients indicates a need for specialized care, which pediatricians believe they are unable to offer. This trend aligns with the global shortage of pediatric rheumatologists, as previously documented in studies from HRIC and MRIC regions [5-9]. Notably, the pediatric workforce in the UAE is similarly constrained, further amplifying the reliance on referrals to specialists rather than first-hand management by general pediatricians.

The diverse training backgrounds of SKMC pediatricians, many of whom were trained outside of the UAE, are likely to lead to varying degrees of PR exposure throughout their residency. For example, pediatricians who completed residencies in regions with more robust PR training infrastructure may have had greater exposure to rheumatic diseases compared to those trained in countries with fewer dedicated programs. Previous studies from Southeast Asia and Asia-Pacific regions [10] have similarly highlighted the uneven PR training across countries, even within regions facing similar healthcare challenges. A standardized PR training program at SKMC could, therefore, address this knowledge gap and equip pediatricians with the necessary skills to improve early detection and management of PR cases, a crucial step in reducing the need for external referrals.

Interestingly, whereas nearly half of the participants reported having had PR training in previous jobs, this did not transfer into high levels of confidence in PR treatment. This gap shows the potential shortcomings of previous training programs, which may have been either too brief or not sufficiently integrated into clinical practice, and thus pediatricians receive only cursory exposure to PR during their training, leading to insufficient practical competence. The majority of residents' willingness to engage in a PR training program demonstrates a significant interest in and understanding of the value of specialized training in this profession.

Additionally, the lack of confidence in managing PR cases reflects a broader issue in pediatric healthcare in under-resourced regions, where many pediatricians are forced to manage complex cases without adequate training or support from specialists. The pediatric gait arms, legs, and spine (pGALS) assessment protocol is a free resource that can support pediatricians who either aren’t PR-specific or do not commonly encounter PR patients [11]. This basic examination of the musculoskeletal system of these juvenile patients gives medical reasoning for both typical and atypical symptoms/presentations.

Several limitations must be acknowledged in this study. Firstly, the survey was conducted exclusively at SKMC, Abu Dhabi, potentially limiting the generalizability of the findings to pediatricians working in other healthcare settings, both within and outside the UAE. Future studies should aim to expand the geographic scope of the survey to include a wider range of institutions, thus providing a more representative multicentric sample. Secondly, the relatively small sample size poses a challenge to interpreting the data with statistical confidence. While the insights gained are valuable, a larger sample would be necessary to draw more definitive conclusions about the preparedness of pediatricians in the region. Thirdly, selection bias is likely present, as non-pediatricians were excluded from participation, limiting the external validity of the study to pediatricians alone. The inclusion of healthcare professionals from other disciplines who may also encounter PR patients, such as orthopedic surgeons or general practitioners, could provide a more comprehensive understanding of the gaps in PR care.

Longitudinal studies assessing pediatricians' performance before and after completing PR-focused education would offer valuable insights into the effectiveness of such programs. Additionally, expanding this research to investigate other potential barriers to PR care, such as access to medications and diagnostic tools, would help to address the broader challenges faced by healthcare providers in treating rheumatic diseases.

## Conclusions

The findings from this survey indicate a clear need for a structured PR training program at SKMC, Abu Dhabi. The majority of pediatricians surveyed lack confidence in examining, investigating, and managing pediatric patients with rheumatic diseases, highlighting a significant gap in PR care. Establishing a comprehensive PR training program at SKMC has the potential to address this gap and improve the quality of care for children with rheumatic diseases. We hope this survey serves as a blueprint for other healthcare institutions in the region facing similar challenges and is the initial step toward further collaborative efforts to improve the quality of PR care in the region.
